# Cisplatin vestibulotoxicity: a current review

**DOI:** 10.3389/fsurg.2024.1437468

**Published:** 2024-10-03

**Authors:** Tamara Fleihan, Marc Elie Nader, J. David Dickman

**Affiliations:** ^1^ Department of Neuroscience, Baylor College of Medicine, Houston, TX, United States; ^2^ Department of Head and Neck Surgery, Division of Surgery, University of Texas MD Anderson Cancer Center, Houston, TX, United States

**Keywords:** vestibular, oncology, neurotology, cisplatin, ototoxicity

## Abstract

Cisplatin, a commonly used chemotherapy drug, is well-established for its ototoxic effects, primarily attributed to the damage it inflicts on cochlear hair cells. However, its impact on the vestibular system remains inadequately understood. Here, we provide a comprehensive review of existing literature concerning cisplatin-induced vestibulotoxicity. Animal studies have shown that cisplatin induces a vestibular hair cell loss that is dose-dependent, with the severity of damage also varying according to the route of administration. Notably, intratympanic and systemic injections in animal models have manifested significant damage primarily to utricular hair cells, with a lesser degree of damage observed for the other vestibular end organs. The underlying mechanisms of cisplatin induced vestibular hair cell loss include apoptosis, oxidative stress, and inflammatory cytokines. Several protective agents, such as Pifithrin-α, DAPT, Ginkgolide B, and heat shock proteins, have demonstrated efficacy in inhibiting cisplatin-induced vestibular damage in preclinical studies. Human clinical findings indicate that cisplatin treatment can cause vestibular dysfunction, characterized by symptoms ranging from transient dizziness to persistent vertigo. Challenges in diagnosis, including the limited utilization of comprehensive vestibular testing for many patients, contribute to the variability in reported outcomes. Cisplatin-induced vestibulotoxicity is a significant complication of chemotherapy, necessitating further research to understand its mechanisms and to improve diagnosis and management, ultimately aiming to enhance the quality of life for cancer patients undergoing cisplatin therapy.

## 1 Introduction

Cisplatin, a potent chemotherapeutic agent containing platinum, stands as a cornerstone in the treatment of various solid tumors due to its remarkable antineoplastic efficacy. However, its clinical utility is limited by significant adverse effects, including nephrotoxicity, neurotoxicity, ototoxicity, and vestibulotoxicity (1).

While extensive research has focused on the ototoxic effects of cisplatin, particularly regarding auditory dysfunction, its impact on the vestibular system remains relatively understudied, creating a notable gap in our knowledge (2, 3). Studies in guinea pigs and rats have demonstrated vestibulotoxic effects, such as hair cell death and a decreased vestibulo-ocular reflex (VOR) gain (4–15). However, the translation of these findings to human clinical scenarios remains elusive. Disturbances in vestibular function with cisplatin therapy can profoundly affect patients' overall well-being. These symptoms are broad, but include balance disorders, dizziness, autonomic disorders, visual instability, and spatial disorientation, highlighting the need for a more comprehensive exploration of cisplatin-induced vestibulotoxicity (16, 17).

The present scoping review aims to characterize the known aspects of complex vestibular dysfunction caused by cisplatin, as well as lay the groundwork for future studies. The primary research questions guiding this exploration are: How do cisplatin treatment regimens adversely affect vestibular function, are these effects long-lasting, and how do vestibular healthcare professionals perceive and navigate cisplatin-induced vestibulotoxicity?

## 2 Methods

### 2.1 Inclusion criteria

The review encompasses studies on cisplatin-induced vestibulotoxicity, from animal models to human studies, ensuring a holistic understanding. A comprehensive search strategy was employed to retrieve relevant peer-reviewed reports in English from two databases, PubMed and ScienceDirect. Excluded were studies published in languages other than English and those that were not peer-reviewed. The search strategy involved a two-step approach: first, we searched for keywords covering cisplatin's ototoxic effects for both the auditory and vestibular systems, and second, we extracted the relevant representations of vestibular dysfunction from these reports. Our search strategy was implemented to ensure a thorough exploration of the literature on cisplatin ototoxicity so that all known vestibulotoxic effects could be documented, even if only briefly mentioned in a specific paper. Key search terms included “Cisplatin ototoxicity” and “Cisplatin vestibulotoxicity”, delving into its effects on the vestibular system specifically. The term “Cisplatin AND vestibular” was employed to capture studies investigating the intersection of Cisplatin and the vestibular system. Other terms associated with the vestibular system function such as vestibuloocular reflex (VOR), head impulse test (HIT), benign positional vertigo (BPV), and vestibular evoked myogenic potential (VEMP) were also used so that the search terms “inner ear”, “tinnitus”, “ vertigo”, “cisplatin AND VOR”, “cisplatin AND HIT”, “cisplatin AND gaze holding”,” “cisplatin AND BPPV”, “cisplatin AND orthostatic hypotension”, “cisplatin AND dizziness”, “cisplatin AND VEMP”, “cisplatin AND oscillopsia”, “cisplatin AND caloric”, were used to ensure a thorough search. Our search strategy aimed to uncover a broad spectrum of relevant literature that would provide a current foundation of knowledge regarding Cisplatin-induced vestibular dysfunction. A total of 38 studies were included.

### 2.2 Data extraction

The methodology involved extracting quantitative outcomes, qualitative insights, and clinical observations from each study to understand cisplatin-induced vestibulotoxicity. Two reviewers analyzed the studies to identify quantitative outcomes and clinical observations. A comparative analysis was then conducted to identify patterns, contradictions, and overarching themes across studies. For empirical studies, relationships between cisplatin dosage and specific vestibular anatomical or functional damage mechanisms were characterized, as well as any variability in responses and assessment methods.

### 2.3 Quality assessment

All studies included in the review had undergone rigorous peer review for reliability and transparency and were obtained from reputable scientific journals. We systematically evaluated each study based on its relevance, validity, and applicability. Additionally, biases and limitations within individual studies were critically considered to ensure a balanced interpretation of findings.

## 3 Results

### 3.1 Animal studies

#### 3.1.1 Vestibular neuroanatomy

It was surprising to discover that relative few animal studies have examined cisplatin-induced vestibulotoxicity. Instead, most of the research has focused upon the ototoxic effects in the auditory system (18, 19). Of the existing literature on vestibulotoxicity, most work has focused on investigating the anatomical effects of ototoxic reactions in vestibular receptors (Table 1). Results from systemic administration of cisplatin have been mixed, with differences noted between damage resulting in the semicircular canal cristae vs. otolith maculae. Not surprisingly, differences in vestibular receptor damage are correlated to the type of cisplatin administration and the dosage used. For example, Nakayama et al. (20) reported targeted vestibular hair cell (HC) loss following a single subcutaneous (SC) injection of cisplatin. Their study in guinea pigs showed that following the administration of a single SC cisplatin injection of 5 mg/kg, the number of hair bundles in the lateral semicircular canal decreased by 21%. The decrease was observed on the central apex portion of the cristae, while the remaining areas of the cristae exhibited hair bundle numbers similar to control (20). Little to no HC loss was observed in the otolith utricular maculae. In rats, Tian et al. (21) observed a significantly higher loss of nearly 60% macular HCs following a single high dose intraperitoneal (IP) injection of 15 mg/kg cisplatin. These findings were aligned with Sergi et al.'s results when IP injections were used as a route of administration in guinea pigs. Following a daily treatment of IP 2.5 mg/kg cisplatin for six days, a moderate reduction in HCs were observed in both the horizontal semicircular canal cristae and utricular maculae (11). In the horizontal cristae, 15% of type I HCs were lost. Still other HCs appeared morphologically abnormal as defined by a loss of calyceal afferent terminals and the appearance of globular degenerating vesicles above the basal laminae (11). In mice, Jiang et al. (6) described a 60%–70% reduction in utricular HCs following daily IP injections of 3 mg/kg cisplatin for 7 days. Alternatively, Fernandez et al. (18) reported no reduction in utricular maculae HCs following a low dose, multi-cycle protocol that is similar to paradigms used to treat human gynecological cancers. These authors examined only the utricle in mice following a multi-week 3 cycle regime of 2.5–3.5 mg/kg IP given once daily for four days, followed by a 10 day rest period. It is unknown if any HC loss occurred in the semicircular canal cristae in these animals.

**Table 1:** Animal models examining cisplatin vestibulotoxicity.

Study	Animal model	Dosage/Route	Findings
Nakayama et al. (20)	Guinea pigs	Single SC injection (5 mg/kg)	21% hair bundle loss in the lateral semicircular canal; little to no HC loss in otolith utricular maculae
Sergi et al. (11)	Guinea pigs	Daily IP injections (2.5 mg/kg) for 6 days	15% type I HC loss in horizontal semicircular canal cristae and morphologically abnormal HCs. Moderate reduction in utricular maculae HCs and VOR.
Lo et al. (8)	Guinea pigs	Daily IP administration (5 mg/kg) for 7 days	Reduced amplitudes in oVEMP responses (5 Hz)
Tian et al. (21)	Rats	Single IP injection (15 mg/kg)	−60% macular HC loss
			20%–35% decline in hVOR and vVOR gain
Callejo et al. (4)	Rats	Intratympanic injection of high dose (2.0 mg/ml)	50%–80% loss of utricular HCs after 7 days
		Intratympanic injection of low dose (0.5–0.75 mg/ml)	Little utricular HCs loss
Rachel et al. (22)	Hamsters	Varying IP doses (7.5, 11.25, 15 mg/ml)	Dose-dependent increase in spontaneous activity (SA) in the dorsal cochlear nucleus
Jiang et al. (6)	Mice	Daily IP injections (3 mg/kg) for 7 days	60%–70% reduction in utricular HCs
Takimoto et al. (13)	Mice	2 or 4 mg/kg i.p daily for 4 days	Significantly reduced 2.5 Hz high frequency VOR response following 4 mg/kg cisplatin given daily for four days. No reduction in VOR gain at lower rotation frequencies of 0.5 or 1.0 Hz.
Fernandez et al. (18)	Mice	Low dose, multi-cycle protocol (3 × 2.5–3.5 mg/kg IP daily for 4 days, followed by a 10-day rest period)	No reduction in utricular maculae HCs
			No significant changes in VsEP responses
Ding et al. (5)	Rat vestibular organ culture	0, 10, 50, 100, 400, or 1,000 µM cisplatin in culture medium for 48 h	U-shaped pattern in HC loss, maximal loss around 50 µM.
Monzack et al. (23)	Mice utricle culture	30 µg/mL cisplatin in culture medium for 48 h	50% HCs loss and 50% supporting cell loss. Impaired scar formation over damaged HCs.
Slattery et al. (14)	Mice utricle culture	5, 10 or 20 µM cisplatin in culture medium for 24 h	Damage to resident stem cells, smaller and fewer spheres.
Slattery and Warchol (24)	Chick utricular culture	20 µM cisplatin in culture medium for 48 h	Decrease in hair cell density by 36% in extrastriolar region; 43% in striolar regions

Individual studies listed by species, cisplatin delivery methods used, and the resulting observations.

Although not typically employed as a clinical strategy for cancer treatment, intratympanic injections of cisplatin have also been used to examine vestibulotoxicity (Table 1). Callejo and colleagues employed intratympanic injections in rats and described that the severity of vestibular toxicity was dose dependent. These authors only examined the otolithic utricular maculae, but observed that a large loss of HC occurred following a single administration of a high dose of 1.0 or 2.0 mg/ml of cisplatin, while little hair cell loss was observed at the lowest doses of 0.5 and 0.75 mg/ml. Based upon the type of cellular marker used to identify HCs [phalloidin or myosin VIIA (MYO7A), a molecular actin binding protein], these authors described between a 50%–80% loss of utricular HCs 7 days after 2.0 mg/ml cisplatin injections. Similar findings were described three days after injection (4) (Table 1).

In addition to *in vivo* studies, organ culture approaches have been used to examine cisplatin vestibular ototoxicity (Table 1). Rat vestibular organ cultures were exposed to cisplatin directly by treating the vestibular explants with 0, 10, 50, 100, 400, or 1,000 μM cisplatin in a culture medium for 48 h (5). A dose-response relationship was identified where a U-shaped pattern in HC loss was described, with maximum hair cell loss around 50 μM (5). Chick utricular cultures were also studied by Slattery et al., where the utricles that were treated with 20 μM of cisplatin for 48hrs showed a decrease in hair cell density by 36% in the extrastriolar regions and by 43% in the striolar regions as compared to the control group (24). In mice, utricles cultured in 30 ug/ml cisplatin experienced a 50% loss of HCs and 50% of supporting cells within 45 h (23). These authors also noted that cisplatin impairs scar formation over damaged hair cells. They suggested that cisplatin inhibits the supporting cell phagocytic removal of dead HCs. It was noted that the accumulation of uncleared apoptotic cells may result in the release of proinflammatory cytokines, leading to secondary necrosis (23).

Cisplatin has also been shown to interfere with the transformation of stem cells into mature HC types (Table 1). Slattery et al. explanted mouse utricles, then treated them with 5, 10, or 20 µM cisplatin for 24 h (14). They next incubated the cells in suspension culture for 3, 5, and 7 days to quantify the number of spheres formed. Results showed that spheres derived from cisplatin-treated samples were smaller and significantly fewer in number compared to the control at all time points. This indicates that cisplatin exposure damages the resident stem cells of the mammalian inner ear. Moreover, it exhibits genotoxic effects even at low doses, targeting hair cells, supporting cells, and epithelial stem cells in the mouse utricle. The study's implications for sensory regeneration are profound, posing challenges for regenerative strategies (14).

A principal finding from these neuroanatomical studies examining the inner ear receptors is that cisplatin elicits a loss of hair cells (Table 1). Through the routes of administration examined so far, cisplatin produces a greater loss of auditory HCs than vestibular hair cells (11, 20). Cisplatin has been shown to gather within the cochlea over an extended period, potentially persisting indefinitely. This accumulation could elucidate its lasting and occasionally delayed impacts on the auditory system. Yet, it remains uncertain whether a comparable phenomenon occurs within the vestibular organs (25). Still, the loss of vestibular hair cells following cisplatin treatment can be profound. This is particularly true for the otolith organs, the utricle and saccule, with smaller deficits observed in the cristae of the semicircular canals (11). Variations in the degree of hair cell loss were associated with different concentrations, doses, and routes of administration. However, few of these studies have used cisplatin in a delivery routine analogous to that used in human clinical cancer treatments. For example, in the treatment of head and neck cancers, cisplatin is administered systemically over a protracted period of time. Each cycle is administered over several hours, with cycles typically spaced three weeks apart. The total number of cycles varies, and common treatment regimens often involve 2–6 cycles. Cisplatin dosage is expressed as mg/m^2^ and often ranges from 40 to 75 mg/m^2^ per cycle with a total dosage reaching up to 200–300 mg/m^2^ (26, 27). Therefore, it is difficult to extrapolate specific findings on vestibular damage from animal model *in vitro* and *in vivo* studies to human subjects.

#### 3.1.2 Vestibular function deficits

Functional vestibular deficits following cisplatin administration have also been examined in animal work, albeit to a much lesser degree (Table 1). The vestibulo-ocular reflex (VOR), a benchmark for examining vestibular efficacy, has been shown to decrease with cisplatin treatment. For example, Sergi et al. (11, 12) examined the VOR in guinea pigs subjected to daily intraperitoneal administration of 2.5 mg/kg cisplatin for six consecutive days (11, 12). They reported a notable 5% reduction in both horizontal (hVOR) and vertical (vVOR) gain during mid-frequency stimulation (0.2–0.4 Hz) and a 10%–15% decrease in the vVOR at lower frequencies (below 0.1 Hz) on the third day of treatment. By the sixth day of treatment, the effects intensified with a 20%–35% decline in hVOR and vVOR gain across the frequency band tested (12). In mice, Takimoto et al. observed a significantly reduced 2.5 Hz high frequency VOR response following 4 mg/kg i.p. cisplatin given daily for four days. No reduction in VOR gain at lower rotation frequencies of 0.5 or 1.0 Hz, nor following a lower dose of 2 mg/kg i.p. cisplatin administration were noted (13). In a similar study, Lo et al. (8) treated guinea pigs with a higher dosage of 5 mg/kg cisplatin daily for seven days and found reduced amplitudes in ocular vestibular-evoked myogenic potential (oVEMP) responses (5 Hz) as compared to saline-treated controls. However, they reported no significant changes in cervical vestibular-evoked myogenic potentials (cVEMP) responses (8). Similar findings of no effect upon the vestibular evoked potential (VsEP) examining otolithic responses were observed in mice following a low dosage treatment of 3 cycles of 2.5–3.5 mg/kg × 4 days followed by a 10 day rest period in mice (18).

To date, no studies have examined vestibular afferents' neural response properties during or following cisplatin administration (Table 1). However, the impact of cisplatin on spontaneous neural activity within the hamster dorsal cochlear nucleus has been examined (22). The findings unveiled a dose-dependent increase in spontaneous activity (SA). Following treatment, the mean peak rate of SA was as follows: 28 spikes/s in the high-dose cisplatin group (15 mg/ml IP), 32 spikes/s in the intermediate dose group (11.25 mg/ml IP), and 29 spikes/s in the low dose group (7.5 mg/ml IP), compared to 14, 15, and 13 spikes/s in the respective control groups (22). It is known that some patients undergoing cisplatin therapy experience increased episodes of tinnitus (28). It is possible that the hyperactivity seen in the cochlear nuclei, which is weighted toward the medial or high frequency region of the dorsal cochlear nucleus (DCN), could underlie the elevated tinnitus. This observation suggests that further investigations are warranted into the intricate interplay between cisplatin, neural activity, and the manifestation of auditory-related complications. This neurophysiological aspect emphasizes the need for a holistic approach to mitigate the vestibular damage induced by cisplatin and the associated neural complications that can significantly impact the patient's quality of life (22).

#### 3.1.3 Mechanisms of vestibular loss

Although it is clear that cisplatin can produce vestibular hair cell damage and loss, the mechanism for such injury remains poorly understood. Several studies have attempted to address this question (Table 2). For example, it is known that many forms of HC death result from apoptosis (7). As noted above, Slattery et al. (24) found that chick utricular maculae exhibited extensive HC loss in cisplatin-treated cultures. They demonstrated that HC death was produced by apoptosis using immunolabeling activated caspase-3 (24). Following such results, it has been shown that agents that affect the apoptotic pathway may provide some protection from cisplatin-induced damage. For example, Pifithrin-α (PFT), known for suppressing p53, a crucial protein in the apoptotic cascade, slowed hair cell loss when administered in conjunction with cisplatin (31). PFT effectively suppresses the activation of caspase-1 and caspase-3, critical components in the apoptotic pathway. These findings emphasized the importance of p53 in cisplatin-induced apoptosis and positioned PFT as a potential therapeutic agent for protection against cisplatin-induced vestibulotoxicity (31). Another approach to protecting hair cells from cisplatin ototoxicity is to manipulate Notch signaling. Notch is known to induce hair cell formation through lateral inhibition and is involved in hair cell proliferation (6). In a recent study, a γ-secretase inhibitor known for modulating Notch signaling {DAPT - N-[N-(3, 5-difluorophenacetyl)-l-alanyl]-s-phenylglycinet-butyl ester}, was used together with cisplatin administration, and the effects on animal swimming test scores (a measure of vestibular function) and preservation of vestibular hair cells were examined. The results following administration of 10 mg/kg of DAPT with 3 mg/kg of cisplatin IP daily for seven days in mice showed that preserved morphologically normal hair cell bundles were present in the vestibular receptors. In contrast, mice treated with cisplatin alone had a significant reduction (−70%) in the number of hair cell bundles as well as disorganized stereocilia (6). In addition, the mice treated with cisplatin alone exhibited vestibular dysfunction manifested by an inability to keep their head above water and circling behavior in a swim test. Results from this study suggest a potential strategy for protecting against vestibular damage induced by cisplatin by inhibiting the Notch signaling pathway (6).

**Table 2:** Animal models examining mechanisms of vestibulotoxicity.

Study	Animal model	Mechanism/Agent	Findings
Sergi et al. (12)	Guinea pigs	Tiopronin	Reduces vestibular HC loss and preserves VOR gains
Lo et al. (8)	Guinea pigs	D-methionine (D-met)	Attenuates decreased VEMP amplitudes and oxidative stress
Ma et al. (9)	Rats	Ginkgolide B (GB)	Reduces vestibular dysfunction by inhibiting caspase activities and enhancing Nrf2-regulated HO-1 antioxidant defense
Tian et al. (21)	Rats	Renexin (RXN)	Protects against cisplatin vestibulotoxicity by preserving hair cells and normal vestibular function
Kim et al. (29)	Mice	Proinflammatory cytokines	Increases TNF-α, IL-1β, IL-6, and NF-κB activation
Baker et al. (30)	Mice	Heat shock proteins (HSP70, HO-1)	Provides partial protection against cisplatin-induced HC death
Jiang et al. (6)	Mice	DAPT (Notch signaling modulator)	Preserves vestibular HCs and reduces vestibular dysfunction
Zhang et al. (31)	Rat vestibular organs	Pifithrin-α (PFT)	Slows HC loss by suppressing p53

Studies listed by species, delivery methods used, and reported findings related to underlying mechanisms of vestibular damage.

A separate investigation examined Ginkgolide B (GB), which serves as an inhibitor of caspase activities and mitochondrial apoptosis, as a potential agent for preserving vestibular function during cisplatin treatment (9). Rats were subjected to intraperitoneal injections of cisplatin (16 mg/kg) and GB (10 mg/kg), either alone or in combination with Zinc Protoporphyrin (ZnPP), a known inhibitor of nuclear factor erythroid 2-related factor 2 (Nrf2), or LY294002, an inhibitor of the survival and growth protein kinase B (Akt) signaling pathway implicated in Nrf2 activation (9). Compared to rats treated solely with cisplatin, those administered with cisplatin plus GB exhibited decreased head rotations during the tail-hanging test and reduced time intervals to complete a swim test. However, when ZnPP or LY294002 was co-administered with GB, the protective effects exerted by GB on vestibular function were not observed (9). Moreover, GB demonstrated inhibition of cellular and mitochondrial reactive oxygen species generation while enhancing Nrf2-regulated HO-1 antioxidant defense in cells exposed to cisplatin (9).

An extensive investigation into the protective effects of renexin (RXN), a combination of Ginkgo biloba extract and Cilostazol, against cisplatin-induced vestibulotoxicity was recently performed using rats (21) (Table 2). A single high dose of 16 mg/kg intraperitoneal cisplatin was used to induce damage. As described above, in the cisplatin-only group, the number of otolith organ hair cells was significantly reduced, with an uneven distribution and abnormal appearance compared to the control group (21). In contrast, the RXN (180 mg/kg) + cisplatin group exhibited well-preserved hair cells with normal-shaped stereocilia and a typical density level. In addition, animals were tested for behavioral vestibular function by examining head rotations and swimming ability. All experimental groups, except for the RXN (180 mg/kg) + cisplatin group, exhibited significantly increased head rotations and prolonged swimming time after five days of treatment. These findings highlighted a role for RXN's protective effect against cisplatin vestibulotoxicity (21).

Tiopronin [N-(2-mercaptopropionyl)-glycerine] is a drug with a free thiol group used in the treatment of hepatic and skin disorders and has been shown to protect against cisplatin-induced nephrotoxicity (12). The drug was also investigated for its vestibular protection by examining vestibular receptor damage and the VOR response (Table 2). It was shown that guinea pigs injected with daily cisplatin (2.5 mg/kg IP) as well as tiopronin (300 mg/kg IP) for six days had lower extent of vestibular hair cell loss in macular and semicircular canals compared to the control group (cisplatin 2.5 mg/kg IP alone). In addition, the VOR gains at 0.05–0.4 Hz were reduced in the cisplatin alone group as compared to the tiopronin + cisplatin group (12). On a histological level, the neuroepithelium of the labyrinth was better preserved (12).

Amino acid-based protectants, such as D-methionine (D-met), have also been employed as a protective agent against cisplatin-induced vestibulotoxicity (Table 2). For example, in guinea pigs, enzyme activities, lipid peroxidation, and VEMP were examined with/without D-met addition during cisplatin treatment (8). When 30 mg/kg IP D-met pre-treatment was given 30 min prior to 5 mg/kg IP cisplatin treatment daily for seven days, the decreased VEMP amplitudes observed with cisplatin alone were attenuated. The authors suggested that this effect was associated with a partial reversal in oxidative stress produced by cisplatin. They found that the cisplatin-only group had the lowest mean Na+, K + - Adenosine 5′-TriPhosphatase (ATPase), and Ca2+-ATPase and the highest lactoperoxidase (LPO) and nitrous oxide (NO) levels as compared to the D-met + cisplatin treatment animals (8).

Outside of the apoptotic pathway and oxidative stress, proinflammatory cytokines, including tumor necrosis factor alpha (TNF-α), interleukin 1 beta (IL-1β), and interleukin 6 (IL-6), have been implicated in cisplatin-induced vestibular cell death (Table 2). These inflammatory markers have been observed to increase in the semicircular cristae and utricles of mice following intraperitoneal injection of 4 mg/kg cisplatin daily for four days (29). Nuclear factor kappa-B (NF-κB), a critical transcription factor involved in the expression of inflammatory cytokines, is known to be activated by TNFα and IL-1β. It was found to be elevated in both the supporting cells and hair cells of the cristae ampullae and utricles after daily administration of 4 mg/kg intraperitoneal cisplatin for four days, suggesting that cytokines would also be elevated. Moreover, cisplatin induced the nuclear translocation of NF-κB, a pivotal transcription factor in the expression of proinflammatory cytokines, by activating mitogen-activated protein kinases such as extracellular signal-regulated kinase (ERK), c-Jun N-terminal protein kinases (JNK), and a mitogen activated protein kinase (p38) (29) (Table 2).

In addition, heat shock protein-mediated protection has also been studied as a potential defense mechanism for cisplatin-induced vestibulotoxicity (30). Cultured mouse utricles that were heat shocked six hours prior to cisplatin treatment (10–60 µg/ml) had significantly greater cell survival proportional to dosage. The cellular survival was present in wild-type mice and absent in heat shock protein 70 (HSP70) knockout mice, highlighting that HSP70 is necessary for the protective effect of heat shock against cisplatin-induced hair cell death (30). However, HSP70 only provides partial protection. Heme oxygenase 1, another heat-inducible protein, was also shown to have a significant protective effect across the dose-response relationship, with macrophages playing an integral role (30). Both proteins were not found to have a synergistic or additive effect when both were expressed (30) (Table 2).

These protective factors, including RXN, tiopronin, GB and heat shock proteins, have demonstrated their efficacy in safeguarding against cochlear toxicity, highlighting a parallel between cochlear and vestibular hair cell damage and protective strategies (9, 12, 21, 30) (Table 2).

The exploration of these potential protective compounds not only guides targeted interventions for clinical applications but also sets the stage for further research, fostering the development of effective and precise strategies to alleviate the adverse effects of cisplatin on the vestibular system.

Why do cochlear hair cells appear to be more susceptible to cisplatin induced damage than vestibular hair cells? Suzuki and Kaga explored the impact of cisplatin on basement membrane anionic sites (BMAs) in guinea pigs (32). Utilizing electron microscopy to analyze temporal bones, cisplatin observed a reduction in cationic tracer density in the stria vascularis capillary wall, hinting at a reduction in BMAs. However, no change in the capillary or subepithelial basement membrane in the ampullar dark cell area was seen after treatment with cisplatin (32). No change in BMAs was observed beneath the sensory cell area of the ampulla and macula. These findings suggest that the charge barrier in the vestibular labyrinth remains functional soon after a single intravenous infusion of cisplatin (32). The authors emphasized that the establishment of a functional charge barrier in the vestibular labyrinth following cisplatin infusion allows only a few cisplatin molecules to be transported to the vestibular sensory cells and dark cells via the capillary and sub-epithelial basement membranes, potentially explaining the reduced toxicity of cisplatin on the vestibular system (32).

### 3.2 Human clinical findings

Opinions on the severity of vestibular deficits in the human population following cisplatin treatment vary (Table 3). Most of the work regarding possible vestibular dysfunction has been gathered from anecdotal self-reports or questionnaires (17). The most common vestibular symptoms reported following cisplatin exposure include temporary dizziness, unsteadiness, imbalance, and vertigo (2, 3, 16, 17, 33). A study on sixty-five testicular and gynecological cancer patients treated with cisplatin observed “balance symptoms” with an incidence of 17% (2, 3). The balance symptoms included vertigo, benign paroxysmal positional vertigo (BPPV), dizziness, unsteadiness, and falls; where vertigo was the most common (9.2%) (2, 3, 16). Brydøy et al. examined 1,409 testicular cancer survivors (TCS) and reported that 22% of the TCS treated with cisplatin reported persistent vertigo or dizziness (28) (Table 3).

**Table 3:** Human clinical findings of vestibulotoxicity.

Study	Population	Methods	Findings
Prayuenyong et al. (2, 3, 16, 17); Trendowski et al. (33)	Testicular and gynecological cancer patients	Self-reports, questionnaires	Common symptoms: dizziness, unsteadiness, imbalance, vertigo; 17% balance symptoms
Brydøy et al. (28)	Testicular cancer survivors	Self-reports	22% persistent vertigo or dizziness
Kitsigianis et al. (34)	Testicular and pulmonary carcinoma patients	Vestibular autorotation tests (VAT)	Significant VOR deficit at high frequencies
Hülse et al. (35)	Cancer patients undergoing cisplatin treatment	VEMP, vHIT	Reduced oVEMP and cVEMP responses, decline in vHIT gain
Moreno and Belinchon (36)	Cancer patients	vHIT	No change in vHIT for patients with a median cumulative dose of 448.87 mg
Myers et al. (37)	Cancer patients	Rotational VOR	No significant difference in VOR gain/phase values for low frequency head rotations
Kobayashi et al. (38)	Cancer patients	Caloric test	Abnormal caloric test results in 50% of patients
Prayuenyong et al. (2, 3)	Cancer survivors	vHIT	Normal vHIT results
Prayuenyong et al. (17)	Clinicians in the audiovestibular field	Survey	32% believed vestibular effects are common, 52% considered it a possible outcome

Studies listed by cancer population, methods used, and related vestibular loss.

Vestibular dysfunction has also been evaluated following cisplatin treatment using more standard assays, including head impulse test (HIT), VEMP, and rotational VOR (Table 3). For example, Hülse et al. measured VEMP responses in patients undergoing cisplatin treatment during randomized control trials at three crucial time points: before the randomized controlled trial (RCT), six weeks after, and three months after cisplatin therapy (35). These patients were given 80 mg/m^2^ of cisplatin at week 1 and 4 (35). At six weeks and three months following treatment, reproducible oVEMP responses were not obtainable in about a third of the patients; reproducible cVEMP responses were also unachievable in 29.3% of patients three months following RCT (35). Nonreproducible cVEMP and oVEMP suggest an impact on saccular and utricular vestibular pathways, respectively. Furthermore, a decline in HIT gain was noted. Prior to RCT, the median gain during horizontal rightward testing was 1.01 and 1.03 during leftward testing, which subsequently diminished to 0.87 and 0.85, respectively following RCT. This reduction was accompanied by an elevation in the frequency of catch-up saccades executed during each head thrust (35). These findings were observed six weeks after the RCT and persisted for three months, providing evidence of vestibular dysfunction associated with cisplatin treatment that appeared to recover over time likely due to mechanisms of central compensation (35, 39). In contrast, Prayuenyong et al. demonstrated that cancer survivors who underwent standard cisplatin therapy (100–400 mg/m^2^) exhibited normal results in eye movements induced by the head thrust test during HIT testing (2, 3). These authors also reported an absence of corrective saccades during the HIT response (2, 3). Similarly, Moreno and Belinchon reported no change in HIT for patients who received a median cumulative dose of 448.87 mg (36). It is worth highlighting that while HIT boasts high specificity, it may compromise sensitivity, potentially accounting for the disparities between rotational chair VOR and HIT findings.

In terms of the more traditional rotational VOR measures, Myers et al. reported that the VOR for low frequency head rotations spanning 0.01–0.16 Hz was not affected by cisplatin treatment. They observed no significant difference in VOR gain/phase values following cisplatin chemotherapy with mean cumulative dosages to 1,600 mg (37). However, a study by Kitsigianis et al. demonstrated a significant VOR deficit induced by high-frequency rotation. In this study, vestibular autorotation tests (VAT) were utilized to assess any VOR changes at high frequencies among five testicular carcinoma patients receiving six weekly treatments of 60 mg/m^2^ cisplatin, as well as four pulmonary carcinoma patients undergoing eight treatments of 100 mg/m^2^ cisplatin (34). The findings unveiled notable reductions in VOR gains and significant phase lags across head rotation frequencies ranging from 3 to 5 Hz in both groups (34) (Table 3).

In the clinical setting, the bi-thermal caloric test is considered the gold-standard measure of vestibular function. However, this tool has been seldom used to evaluate the impact of cisplatin on vestibular function in human subjects (Table 3). Kobayashi et al. observed an abnormal caloric test in half of the 10 cancer patients treated with 80–550 mg of cisplatin (38) (Table 3). Currently, no study of patients treated with cisplatin integrates the caloric test, rotational chair test, and HIT to evaluate the VOR comprehensively across different frequency ranges in the same setting.

Based upon the complexity of vestibular testing, the degree of such testing performed, and the limited literature on the subject, it is not surprising that a survey distributed among clinicians working in the audiovestibular field showed that 32% of participants believed it often causes such effects, and 52% considered it a possible outcome (17) (Table 3). This lack of consensus within the medical community adds a layer of complexity to the interpretation of vestibular symptoms. Clinicians with a biased opinion that no vestibular deficits result from cisplatin treatment may overlook subtle or complex vestibular involvement (17). It emphasizes the need for a nuanced diagnostic approach to consider a range of symptoms, from subtle balance issues to BPPV and vertigo (2, 3, 16).

The cumulative findings underscore the necessity for advanced diagnostic tools, interdisciplinary collaboration, and continued scientific research to unravel the complexities of cisplatin-induced alterations in auditory and vestibular functions across diverse patient populations. Imaging procedures such as labyrinthine enhancement on 3D black blood MR images monitoring alterations in the labyrinth induced by cisplatin exposure may allow for more precise diagnosis (40). However, these techniques remain understudied in cisplatin vestibulotoxicity, and prospective studies are needed to validate them before they can be used routinely in this condition.

## 4 Discussion

Here, we provide a scoping review of a comprehensive analysis of cisplatin-induced vestibulotoxicity, incorporating insights from animal studies and human clinical findings. The animal studies have illuminated various aspects of vestibular damage caused by cisplatin, including dose-dependent effects, differences in damage between intratympanic and systemic administration, and differences in damage between the semicircular canal cristae and the otolith maculae (4, 5, 8, 11, 12, 18, 23, 24). Additionally, protective factors such as Pifithrin-α, DAPT, Ginkgolide B, Tiopronin, and others have been explored, revealing potential therapeutic avenues (9, 12, 21, 30). However, it is difficult to extrapolate findings from animal studies to human subjects due to differences in delivery and dose protocols of cisplatin. Thus, it is apparent that further research in animal models is prudent particularly in adopting multi-dose cyclic treatment protocols that mimic more closely those being utilized currently in the clinic to treat various forms of human cancer.

The underlying mechanisms of vestibular hair cell loss, including apoptosis, oxidative stress, and inflammatory cytokines have been investigated, providing valuable insights into the pathophysiology of cisplatin-induced vestibulotoxicity (6, 8, 14, 24, 29, 31). However, there are challenges in translating these findings into clinical practice, highlighting the need for further research, and targeted interventions.

The clinical findings pertaining to vestibular deficits following cisplatin treatment in humans are diverse, with discrepancies in the prevalence and severity of symptoms (2, 3, 16). This underscores the pressing need for standardized vestibular assessment protocols and interdisciplinary collaboration to accurately diagnose and manage vestibular dysfunction in patients undergoing cisplatin therapy. For example, many facilities providing cancer treatment have commonly available vestibular dysfunction clinics that offer nystagmography, either electro (eVNG) or video (vVNG) for caloric, positional, or head impulse testing, as well as VEMP testing. Together, these vestibular assessment batteries require approximately 1–2 h of patient time, with subsequent interpretative findings provided to the oncology team. More sophisticated vestibular testing facilities may also have available rotational chair or dynamic posturography equipment that can further pinpoint affected vestibular dysfunction. In cases where vestibular dysfunction from cisplatin treatment is suspected, or in cases where larger cisplatin dose therapy is required, the current review suggests that a standard protocol of at least VNG or VNG combined with VEMP testing be employed so that even subtle vestibular dysfunction findings can be objectively evaluated, with subsequent vestibular rehabilitation therapies employed. This standardized protocol could mirror those for auditory testing and include baseline testing before the start of chemotherapy, testing upon completion of cisplatin therapy, and additional testing before each cycle if new vestibular symptoms emerge.

Future research directions hold immense potential, including a focus on enhancing diagnostic tools, exploring novel therapeutic targets, and improving our comprehension of the complex interplay between cisplatin and the vestibular system.
